# Applying Human-Centered Design in Global Mental Health to Improve Reach Among Underserved Populations in the United States and India

**DOI:** 10.9745/GHSP-D-22-00312

**Published:** 2023-02-28

**Authors:** Candace J. Black, Jenna M. Berent, Udita Joshi, Azaz Khan, Lila Chamlagai, Ritu Shrivastava, Bhuwan Gautam, Abdikadir Negeye, Abdi Nor Iftin, Halimo Ali, Alethea Desrosiers, Anant Bhan, Sunand Bhattacharya, John A. Naslund, Theresa S. Betancourt

**Affiliations:** a Research Program on Children and Adversity, School of Social Work, Boston College, Boston, MA, USA.; b Bangalore Hospice Trust – Karunashraya Institute for Palliative Care Education and Research, Bangalore, India.; c Sangath, Bhopal, India.; d Community member from the resettled Bhutanese community, Springfield, MA, USA.; e Community member from the resettled Somali Bantu community, Lewiston, ME, USA.; f Department of Psychiatry and Human Behavior, Warren Alpert Medical School, Brown University, Providence, RI, USA.; g Boston College School of Social Work, Chestnut Hill, MA, USA.; h Department of Global Health and Social Medicine, Harvard Medical School, Cambridge, MA, USA.

## Abstract

**Introduction:**

Human-centered design (HCD) refers to a diverse suite of interactive processes that engage end users in the development of a desired outcome. We showcase how 2 global mental health research teams applied HCD to develop mobile health tools, each directed at reducing treatment gaps in underserved populations.

**Case Study 1:**

Refugees face higher risks for mental health problems, yet these communities face structural and cultural barriers that reduce access to and use of services. To address these challenges, the Research Program on Children and Adversity at the Boston College School of Social Work, in partnership with resettled refugee communities in the northeastern United States, used codesign methodology to digitally adapt delivery of the Family Strengthening Intervention for Refugees—a program designed to improve mental health and family functioning among resettled families. We describe how codesign methods support the development of more feasible, acceptable, and sustainable interventions.

**Case Study 2:**

Sangath, an NGO in India focused on mental health services research, in partnership with Harvard Medical School, designed and evaluated a digital training program for community health workers to deliver an evidence-based, brief psychological treatment for depression as part of primary care in Madhya Pradesh, India. We describe how HCD was applied to program development and discuss our approach to scaling up training and capacity-building to deliver evidence-based treatment for depression in primary care.

**Implications:**

HCD involves a variety of techniques that can be flexibly adapted to engage end users in the conceptualization, implementation, scale-up, and sustainment of global mental health interventions. Community solutions generated using HCD offer important benefits for key stakeholders. We encourage widespread adoption of HCD within global mental health policy, research, and practice, especially for addressing mental health disparities with underserved populations.

## Abstract (precis)

We demonstrate how 2 global mental health research programs engaged end users to design tailored, culturally informed digital tools used to support the delivery of evidence-based interventions.

## Teaser

**Key Findings:**

- Engaging end users in the development of mental health intervention delivery tools ensured that the design features reflected the perspectives and needs of those who would use them.
- Technology may increase the reach of mental health interventions and offers several potential benefits, including supporting capacity-building in low-resource settings; improving technical literacy in underserved communities; and achieving difficult scale-up processes.

**Key Implications:**

- When developing mHealth tools with underserved populations or those with lower technical literacy, researchers and practitioners should build supportive mechanisms within the development process to help users engage in the design and evaluation of each product’s features.
- Policymakers, national stakeholders, researchers, and practitioners can potentially reduce mental health treatment gaps by recruiting and training lay health workers using task-sharing models.

## INTRODUCTION

Global mental health is a relatively new field directed at achieving mental health equity worldwide.^1^ Driven by the perspective that mental health is a fundamental human right,^2^ global mental health research has sought to reduce the mental health treatment gap—a measure of unmet need in mental health—especially in low- and middle-income countries where it is most pervasive.

In this article, we describe how 2 global mental health research teams have implemented research that targets the care gap—a similar but more holistic concept than the treatment gap that acknowledges the psychosocial and physical health care needs of people with mental health problems. We first introduce human-centered design (HCD) methodology and describe how this particular approach dovetails with the mission and values of global mental health research. We then provide 2 case studies to illustrate how HCD has been applied to enhance the implementation and delivery of evidence-based mental health interventions in the United States and India. The benefits and challenges, lessons learned, and implications for research and practice are discussed.

Global mental health arose from the confluence of several transformational shifts in the social and biomedical sciences centered around self-determination, human dignity, and human rights.^3^ For example, the etiology of mental health disorders evolved from a purely biomedical perspective to one that encompassed social, environmental, and institutional contexts, resulting in a fundamental shift toward more humanistic and holistic mental health interventions. These movements deconstructed the standard model of mental health care, which relied on institutionalization and in which the majority of service provision was conducted by psychiatrists. Instead, the new paradigm incorporated rehabilitation and community care, expanded the range of acceptable qualifications to engage in mental health treatment, and empowered a new generation of mental health stakeholders to become nonspecialist providers, including community health workers (CHWs), educators, caregivers, and the people affected by mental disorders themselves.

The field of global mental health made its debut with a 2007 Lancet Global Mental Health Group call to action to address global mental health disparities by scaling up mental health care, especially in low- and middle-income countries.^4^ Several global initiatives emerged to support significant developments in nonspecialist workforce development, establish standards of care, collect data for tracking progress, and strengthen governance. However, these investments lacked impact on a global scale. Ten years after the initial call to action, the Lancet Commission on Global Mental Health and Sustainable Development was established to align global mental health with sustainable development, thereby leveraging the ambitious vision and widespread momentum of the United Nations Sustainable Development Goals to improve mental health and reduce the overall disease burden worldwide.^2^ The Commission identified 4 features of global mental health interventions that should be prioritized for scale-up efforts (Box).

BOX **The Lancet Commission’s Priorities in Global Mental Health Interventions for Scale-Up^2^**

- Shift foundation of mental health care system to task-sharing models with nonspecialist workers implementing psychosocial interventions.
- Coordinate mental health care with primary and specialist care to improve prevention and physical health gaps which often accompany the mental health treatment gap.
- Adopt digital tools to facilitate intervention delivery.
- Implement community-based interventions to increase service demand and utilization.

## HCD IN GLOBAL MENTAL HEALTH RESEARCH PROGRAMS

We selected 2 global mental health research programs to illustrate how the priorities recommended by the Lancet Commission (Box) are implemented in real-world settings. Both case studies describe efforts to digitally adapt components of evidence-based mental health interventions or their implementation. Both interventions were developed with strong input from the communities in which they have been implemented, and both of them are delivered by nonspecialist CHWs. Importantly, both teams engaged end users in the digital adaptations by using principles of HCD—a powerful and versatile framework for problem-solving and innovation that can be used to address mental health disparities and inequitable access to essential mental health services.^5^ Although HCD approaches vary, core features involve engaging relevant stakeholders and centering their needs in the design process.^6^ HCD has long been recognized for its potential to improve user satisfaction of digital products and services. More recent applications in mental health interventions have shown promise for enhancing effectiveness, efficiency, accessibility, and sustainability of evidence-based treatments.^7^ These positive outcomes are facilitated by HCD’s application of systems thinking, which recognizes interrelations among individuals, groups, and societies. This holistic approach restructures common power dynamics toward equity, engendering trust and collaboration and supporting stakeholder ownership of solutions.^8^

We highlight 2 global mental health interventions that developed delivery tools using HCD principles, integrated strong community input, and are delivered by nonspecialist providers.

The first case study describes the development of a mobile app for resettled refugee families involved in a mental health intervention being tested in the United States. We illustrate how design thinking can be applied at early stages of app development and describe the process of codesigning digital imagery for the app. We also highlight challenges faced as the early stages of codesign occurred during the COVID pandemic. The second case study describes the development, implementation, and evaluation of a digital program for training CHWs to deliver an evidence-based, brief psychological treatment for depression in routine primary care settings in Madhya Pradesh, India. This case study is interesting as an advanced stage demonstration of how HCD was applied in each stage of development, adaptation, and testing; formal evaluation; and implementation and scale-up.

RPCA used a CBPR approach to collaborate with locally resettled refugees to adapt a mental health intervention to address traumatic stress and depressive symptoms.

## CASE STUDY 1: CODESIGNING WITH BHUTANESE AND SOMALI BANTU REFUGEES IN THE UNITED STATES

### Background

Refugee families often experience traumatic stress from persecution, war, and displacement, followed by resettlement and acculturation stress.^9^ Elevated risk for developing mental health problems is compounded by cultural and systemic barriers to accessing mental health services.

The Somali Bantu, an ethnic minority originating in sub-Saharan Africa, faced enslavement and marginalization beginning in the 19th century. Civil war erupted in Somalia in 1991, resulting in forced migration to refugee camps in Kenya, where they were deprived of education, jobs, and statehood. In 2004, more than 13,000 Somali Bantu were resettled in the United States.^10^

In the 1990s, more than 100,000 Lhotshampas Bhutanese were evicted from Bhutan following ethnic cleansing and forced to resettle in refugee camps in Nepal characterized by challenging living conditions and myriad diseases. Starting in 2007, more than 110,000 Bhutanese refugees began resettling in the United States.^11^

### Program Development and Adaptation

The Research Program on Children and Adversity (RPCA), at the Boston College School of Social Work, used a community-based participatory research (CBPR) approach to adapt a mental health intervention for locally resettled Somali Bantu and Lhotshampas Bhutanese refugees in the northeastern United States. The intervention was based on the Family Strengthening Intervention (FSI), which was adapted from the Family-Based Preventive Intervention, and that promotes healthy parenting and mental health in children of HIV/AIDS-affected caregivers in Rwanda.^12^

RPCA researchers cultivated relationships with resettled refugees and collaborated with them on CPBR activities to adapt FSI content to address acculturative stress, leverage community and family resilience, and reduce mental health disparities. The resulting Family Strengthening Intervention for Refugees (FSI-R) follows a home-visiting model, using trained lay CHWs to provide families with psychoeducation, coaching, and skill-building tools through 10 weekly modules. Randomized pilot testing demonstrated feasibility and acceptability and showed promise for reducing traumatic stress and depressive symptoms, as well as conduct problems among youth.^13^

CBPR emphasizes equity among community members, researchers, and stakeholders,^14^ identifying community members as integral team members throughout the research and implementation process. Somali Bantu and Bhutanese community members participated in needs assessments, served on community advisory boards, and were employed as research assistants, CHWs, and supervisors. FSI-R development incorporated community feedback about community-based protective resources such as support from mutual assistance organizations, cultural and religious organizations, and culturally relevant resources to emphasize the unique needs, strengths, and challenges of resettled communities.^9^

FSI-R delivery used paper-based manuals. However, exit interviews with pilot CHWs revealed that families and CHWs could benefit from digitized FSI-R materials to support navigation while also increasing engagement and learning through graphics, culturally relevant imagery, embedded videos, and hyperlinks to additional resources. Boston College supported a multidisciplinary team of designers, student programmers, public health researchers, and industry professionals to guide the HCD and adaptation process of the FSI-R manual into 2 digital FSI-R apps: a CHW-facing version supporting training and delivery and a family-facing version supporting engagement and learning.

Design thinking, including problem analysis, consultations with staff and community advisory boards, and user interface/user experience (UI/UX) testing supported app development. The UI/UX testing incorporated a multimethod approach to better understanding the usability, functionality, and features of the app, including: “think aloud” methodology, whereby the participant is given a set of instructional actions to perform in the app and speaks their thought processes aloud while performing them; video recording of the participant’s movements and navigation throughout the app to better understand speed and ease of use; and an open-ended semistructured interview to elicit additional feedback and user thoughts. All data, including audio and visual recordings, were coded and analyzed for themes. The findings revealed navigational pain points, features that were not well-liked, and symbols or icons that did not present clearly. For example, participants identified difficulty in discerning which icons or images could be clicked on to yield additional information, which resulted in the addition of design features to distinguish interactive icons. The process also highlighted features and functions that were particularly helpful, clear, and well received. For instance, participants revealed that they benefited from the contents on the home screen and that they were able to navigate efficiently throughout the app with the use of arrows.

UI/UX testing findings revealed features that were not well-liked as well as ones that were helpful and well received.

In addition to strengthening UI/UX features of the app, we also applied HCD to incorporate the cultural beliefs of participating communities using visually recognizable elements. We felt that the codesign process was important for this technology-driven intervention for several reasons. First, codesign aligns with RPCA’s CBPR approach and emphasis on intervening “for refugees, by refugees.” Codesign encourages a sense of ownership in the creation of solutions. Second, codesign brings users closer to the visual interface and its functioning by graphically incorporating culturally familiar features that would further support learning about FSI-R concepts. Lastly, codesign can help to reduce “tech anxiety,” which is especially relevant when users lack experience with digital tools.

### Ethical Approval

Community members were recruited into community co-design teams (CCDTs) to enhance cultural relevance of graphic design features in the family-facing app. Ethics approval was obtained from the Boston College Institutional Review Board. All participants gave their informed consent before joining CCDTs.

### Implementation

FSI-R staff recruited 8 adults and youth from each resettled community via a description posted to social media and shared via word-of-mouth. Eligibility criteria included identifying as part of a resettled Bhutanese or Somali Bantu community and aged older than 13 years. The Somali Bantu CCDT was composed of up to 3 male and female adults and up to 4 adolescent girls, while the Bhutanese CCDT was composed of up to 2 male adults and up to 5 adolescent boys and girls. Participants were compensated with US$15 gift cards per session and given a certificate of appreciation upon completion. A community volunteer facilitated each session. RPCA staff included the program manager, a postdoctoral researcher, and a paid student intern.

Due to the COVID pandemic, sessions were conducted virtually using an interactive Google Slides deck and Zoom videoconferencing software. Participants joined by computer or mobile phone and were encouraged to join by video to increase participation. Sessions were 1.5 hours weekly over 10 weeks. RPCA staff led an orientation to introduce the goals of codesign, materials and technology, ground rules, and practice examples. Participant contributions were integrated directly into the shared Google Slides deck for immediate viewing and live interaction. Following each session, a graphic designer used Procreate to create a composite that reflected sample images, text descriptions, and CCDT feedback.

The codesign protocol included the following 6 iterative steps, adapted for remote application.

1. Over a Zoom video conference call, the facilitator presented a Google slide with a written description of an FSI-R concept (e.g., “family strengths”). Any clarifying questions were answered.
2. Facilitators indicated the next several minutes (e.g., 2–5 minutes) would be allotted for CCDT members to independently brainstorm how the FSI-R concept could be best represented visually. CCDT members were asked to choose imagery that would be widely understood by families participating in the FSI-R by thinking about their own experiences, cultural history, and/or values and how the concept might look for a family in their community.
3. On the shared Google slide deck, participants added their thoughts from the brainstorming session directly to an Ideas slide using drawings, Internet images, personal photos, or verbal descriptions. Where bandwidth issues prevented direct interaction with Google slides, facilitators documented ideas following verbal guidance from CCDT members. Once each participant had added their idea(s), facilitators asked each participant to verbally share their rationale for choosing an image. This discussion also allowed facilitators to probe for additional details as needed to ensure key features are represented visually. Where possible, additional images were retrieved and detailed notes were recorded on comments about the concept.
4. Each idea for a visual representation was then placed on virtual notecards on a new Google slide so that all images could be viewed at once. Several star-shaped indicators placed on the slide were selected by participants and moved next to their preferred image. Where bandwidth issues prevented direct interaction, facilitators assisted participants by moving the star indicator next to their preferred image expressed verbally. Participants were asked to consider perspectives of community members characterized by diverse genders, ages, and experiences when choosing an image. The image with the most votes was selected for further discussion and refinements. Occasionally, more than 1 image was highly voted and, where possible, multiple images were retained for further discussion and refinements. Facilitators asked community members what they liked and disliked about the image and whether they would change anything about the image. Detailed notes documented these discussions.
5. The notes and images collected during CCDT meetings were provided to a graphic designer who created an illustrated prototype. Facilitators also provided additional context to guide graphic designers where needed. As these exchanges were also remote due to the COVID pandemic, materials were provided as shared files where facilitators and graphic designers could edit documents in real time. In addition to email, phone, and virtual conference contact with graphic designers, facilitators also provided guidance via comments applied directly in the shared file.
6. The completed prototype was presented to the team during a subsequent meeting or by email, and members were asked for feedback on the prototype. Any additional refinements suggested by the team were provided to the graphic designer, who made adjustments for final use of the image.

**FIGURE 1:**
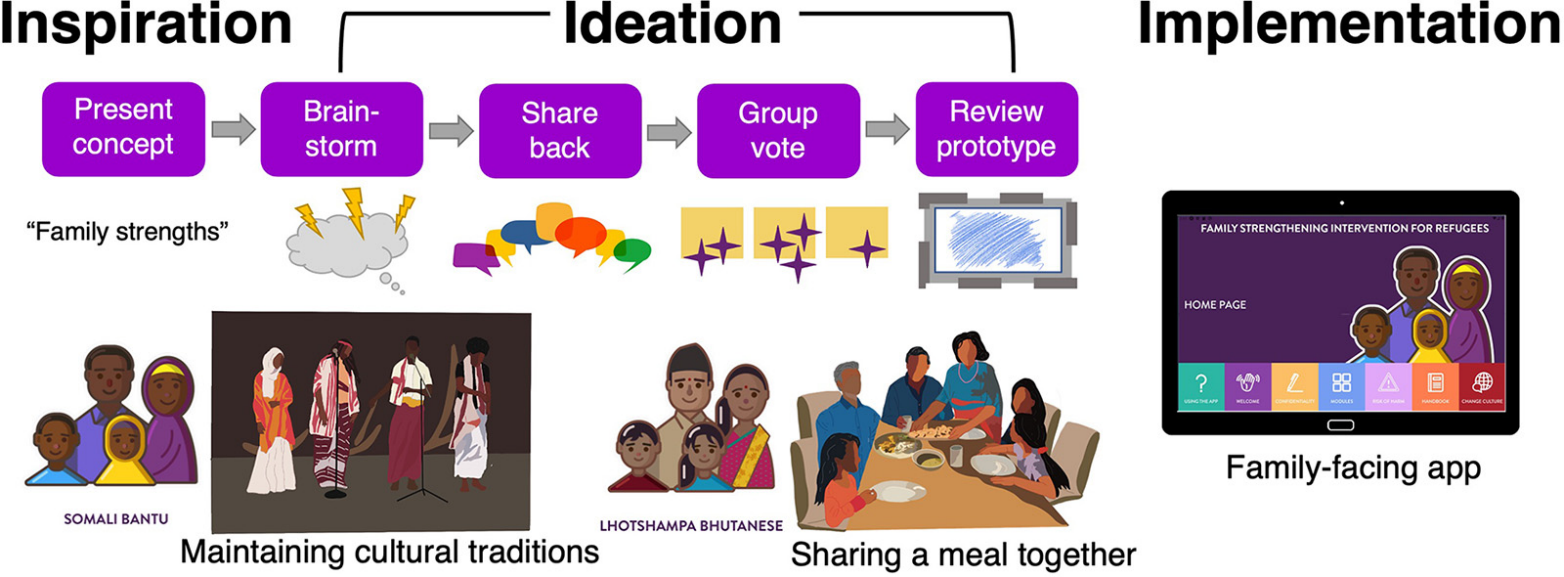
Codesign Protocol Alignment With Inspiration, Ideation, and Implementation Phases of Human-Centered Design

This process was repeated for selected main concepts from the 10 FSI-R modules. Figure 1 displays the alignment of the codesign protocol with the 3 major phases of HCD. The majority of the work completed by following this protocol was directed at the ideation phase.

### Lessons Learned

#### Strengths

**Design features shaped by early-stage testing**. Early UI/UX testing ensured that the app’s design features aligned with perceptions and expectations of end users. For instance, Think Aloud testing revealed how these features were interpreted while navigating the app, which helped identify both strengths and areas for improvement. Conducting UI/UX testing at the earliest stages of app development ensured that all subsequent iterations of the app reflected this feedback.

**High-quality, culturally appropriate imagery developed through codesign**. Our CCDTs were composed of participants recruited from within each resettled refugee community involved in the FSI-R. By conducting codesign with these community members, we were able to develop high-quality images that reflected the cultural values and history of each community. These images will be embedded in the family-facing version of the mobile app, accompanying data-light text descriptions of key ideas covered in each module of the intervention. For instance, the family narrative is a key component of the FSI-R, which asks participants to develop a timeline and narrative of their family’s experiences. Codesign supported development of culturally relevant icons to accompany an example timeline with events commonly experienced by resettled families. Additionally, codesign enabled the team to develop visual imagery to accompany more abstract ideas featured in many of the FSI-R modules, such as “family challenges” and “family strengths.”

**Representation of diverse perspectives and promotion of equity**. Engaging communities in the development of interventions, including tools and auxiliary materials, empowers community agency and autonomy. HCD creates a shared learning environment beneficial for researchers and community members. Our CCDTs included diverse ages and genders, and, in the case of 1 community, diverse caste members. With multiple perspectives, discussions explicitly assessed how images might be made more gender-sensitive and inclusive.

Engaging communities in the development of interventions empowers community agency and autonomy.

**Community buy-in and mutual learning**. Rooting intervention research in CBPR and HCD methods is a bottom-up approach based on mutual learning between communities and researchers. CCDT discussions involved rich cultural histories, practices, and values, as well as lived experiences of team members, enabling the research team to challenge their own biases and produce a more meaningful, better-quality product. The community also benefits from resources and connections with educational institutions, empowered youth, and increased social capital (B. Gautam, oral personal communication, June 2022).

**Improved feedback**. Each iterative feedback loop of our codesign method offered opportunities for input, discussion of concepts, clarification of ideas, retrieval of diverse source material, anonymous voting for images, and opportunities to tailor images through multiple feedback loops. Emphasizing inclusion and openness to member ideas meant that final illustrations accurately represented cultural perspectives.

#### Challenges

**Time constraints**. CCDT discussions generated multiple culturally rich suggestions for imagery and often delved into the histories, beliefs, and values of each community. Occasionally, our efforts to gather information so that all voices were heard posed challenges given the allotted time available for the project. Ultimately, we added sessions to achieve adequate coverage of the topics. Including strategic planning to solidify the scope of work, allotting time in the schedule accordingly, and using a timer could facilitate more timely progress.

**Technological limitations**. CCDT members brought varying levels of experience with technology, and some were learning these technologies for the first time, reflecting the low digital literacy and digital divide experienced by some members of marginalized communities. The orientation session was essential for training CCDTs on the use of these technologies, and ongoing technical support was provided, as needed. Furthermore, Internet connectivity was occasionally a challenge. When necessary, members adapted by turning off video or joining by phone. Moreover, recruiting and conducting the sessions by digital means essentially excluded certain demographics, such as older generations or individuals without WiFi access. Although we asked participants explicitly to consider diverse perspectives when generating imagery ideas as part of the protocol, this approach likely did not fully capture the depth and breadth of alternative views. In asking participants how we might engage more community members, we received feedback suggesting that in-person sessions would likely alleviate technological challenges, increase diversity further, and improve participation.

**Recruitment and retention**. Communities varied in their availability and willingness to participate. For one community, we lacked youth perspectives for several sessions, and some members stopped joining. We partially attribute attendance challenges to the digital format because, before the pandemic, in-person gatherings had greater and more consistent attendance.

## CASE STUDY 2: DIGITAL TRAINING PROGRAM TO DELIVER PSYCHOLOGICAL INTERVENTION FOR DEPRESSION IN RURAL INDIA

### Background

The World Health Organization Mental Health Gap Action Programme recommends brief psychological interventions as a first-line treatment for depression.^15^ However, in low- and- middle-income countries like India, delivery of brief psychological interventions is remarkably challenging because of a scarcity of specialist providers to deliver these treatments, supervise care, or train additional therapists.^16^^,^^17^ These concerns are further exacerbated in rural areas, where upwards of 90% of individuals living with depression and other common mental disorders do not receive adequate care.^18^ Consequently, mental health capacity-building is a priority in India and for extending global mental health efforts more broadly.^19^

Approaches like task-sharing support capacity-building of health workers^20^^,^^21^ who can effectively deliver a brief psychological intervention. Thus, task-sharing holds potential to address the care gap for depression^22^^,^^23^; however, a key bottleneck to effective implementation is the need to train these health workers to deliver these psychological treatments and to ensure that this workforce achieves the necessary clinical competencies to deliver high-quality depression care.^24^^,^^25^

Traditional health worker training methods, like in-person training, are most common both in India and globally. However, the need for expert trainers, access to training facilities, and frequently required extensive travel for participants pose financial and logistical barriers to scalability.^16^ Increasing access to and use of digital technologies among health workers may offer opportunities to leverage these technologies to expand capacity remotely.^26^ For instance, digital technology can enhance in-person training programs for health workers in low-resource settings by offering remote support, allowing asynchronous access to training materials and content, and enabling learning tracking and monitoring progress.^26^^,^^27^ To build on this promise, this case study involved the systematic and iterative development of a digital training program to scale up efforts to build capacity of CHWs to deliver depression care in rural India.^28^

This case study involved the development of a digital training program to build CHWs’ capacity to scale up depression care in rural India.

### Program Development and Adaptation

**FIGURE 2:**
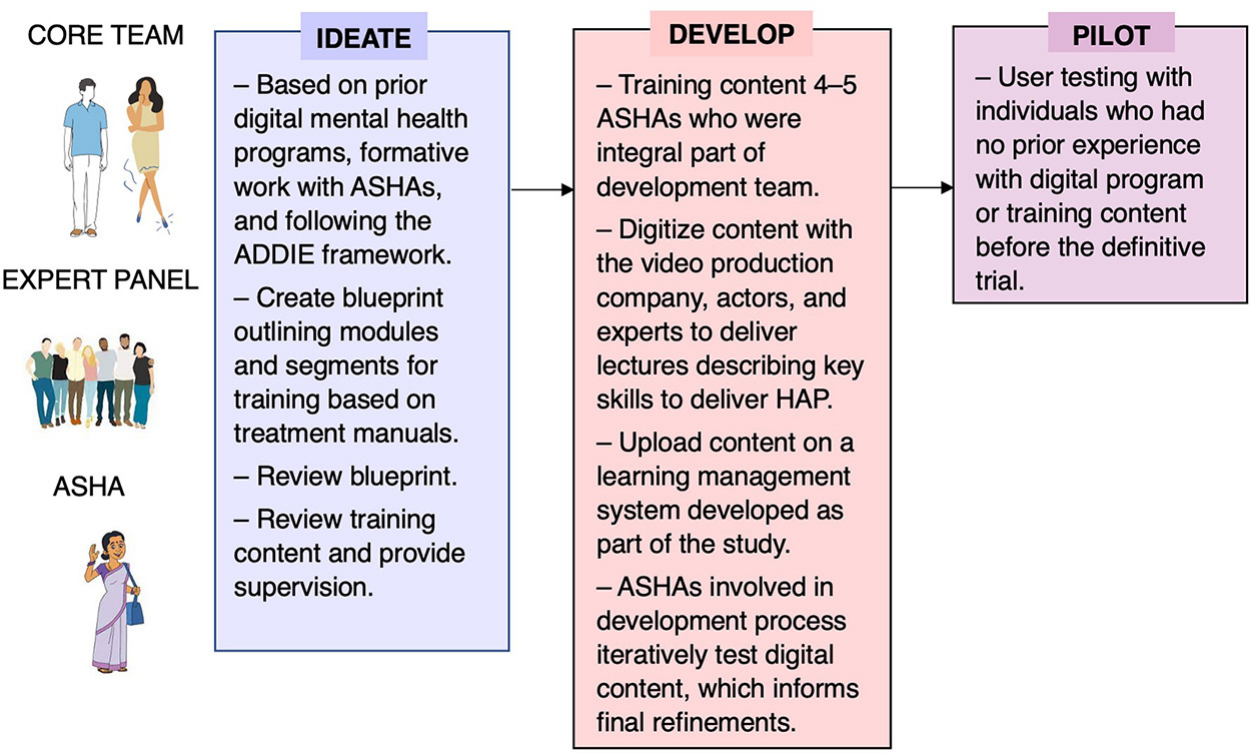
Iterative Process of Codesign Consultation for Development of the Training Program For a Brief Psychological Intervention for Depression in Rural India Abbreviations: ADDIE, Analysis, Design, Development, Implementation, and Evaluation; ASHA, accredited social health activist; HAP, Healthy Activity Program.

The development of the digital training program began with a core team of researchers who had previously supported the development and evaluation of the Healthy Activity Program (HAP), an evidence-based, brief psychological treatment for depression based on behavioral activation and adapted for use in Goa, India.^29^ The study employed HCD and involved collaboration with accredited social health activists (ASHAs), a cadre of trained CHWs who work with the National Health Mission to deliver essential routine primary care services to rural areas and promote community utilization of the existing health services.^30^^,^^31^ With ASHAs as the target audience of our training efforts, it was critical to ensure that these health workers were engaged throughout the development and pilot testing of the digital training program, practices that are integral to HCD. We used an iterative design approach to develop the digital training program, initiated first through formative research activities with ASHAs in the form of focus group discussions and design workshops to better understand their perspectives and feedback on the training content and potential for using digital technology in its delivery.^20^ Our work was also guided by prior digital mental health initiatives,^32^ and informed by the ADDIE (Analysis, Design, Development, Implementation, and Evaluation) framework^33^ with a focus on learners’ engagement as well as cultural and contextual adaptation.^34^ Our systematic approach, illustrated in Figure 2, involved 5 key steps.

**1. Create blueprint of training program**. We conducted a careful review of the original HAP treatment manuals so that the outline of the digital training content would retain fidelity to the core components of the manuals. This involved multiple rounds of expert review with psychologists and counselors with experience delivering psychological treatments and training health workers in settings in India.

**2. Develop training program content**. Prior formative research activities in which ASHAs expressed their preference for video-based content and use of visuals to effectively reflect realistic scenarios guided us in the development of digital content and representation of the local culture and context.^20^ For content development, we engaged 4–5 ASHAs (age range: 18–40 years) in a brainstorming session to discuss the character and stories for role-play, colloquial language, and terminologies. Based on the discussions, HAP content was adapted and converted into scripts, which included local montages, translation into local language, and simplification of complex terms. The content also included scripted role-plays of ASHAs delivering HAP to patients in health care and community settings. The same group of ASHAs reviewed the translation of the materials and ensured that the content was relevant for use in the target setting. Throughout this process of digital training content development and script creation, counselors with expertise in the delivery of HAP reviewed the content to ensure ongoing fidelity to the evidence-based HAP intervention.

**3. Digitize training program content**. Based on the ASHAs’ preference for video-based content, we worked with professional video production companies to develop short films that included lectures by experienced counselors on the key skills for delivery of HAP and role-play scenarios illustrating application of the skills. The video-based content was supplemented with PowerPoint-based lecture videos and reading materials, which were simpler for our team to develop and also easier to modify as needed, as well as culturally relevant graphics. This process involved multiple rounds of editing and review from our team to ensure the quality of the digital content and fidelity to the original evidence-based HAP program. The ASHAs involved in Step 2 iteratively tested the digital content. Their feedback and comments primarily focused on the cultural adaptation of the content by including local images, places, and culturally relevant phrases.^20^

**FIGURE 3:**
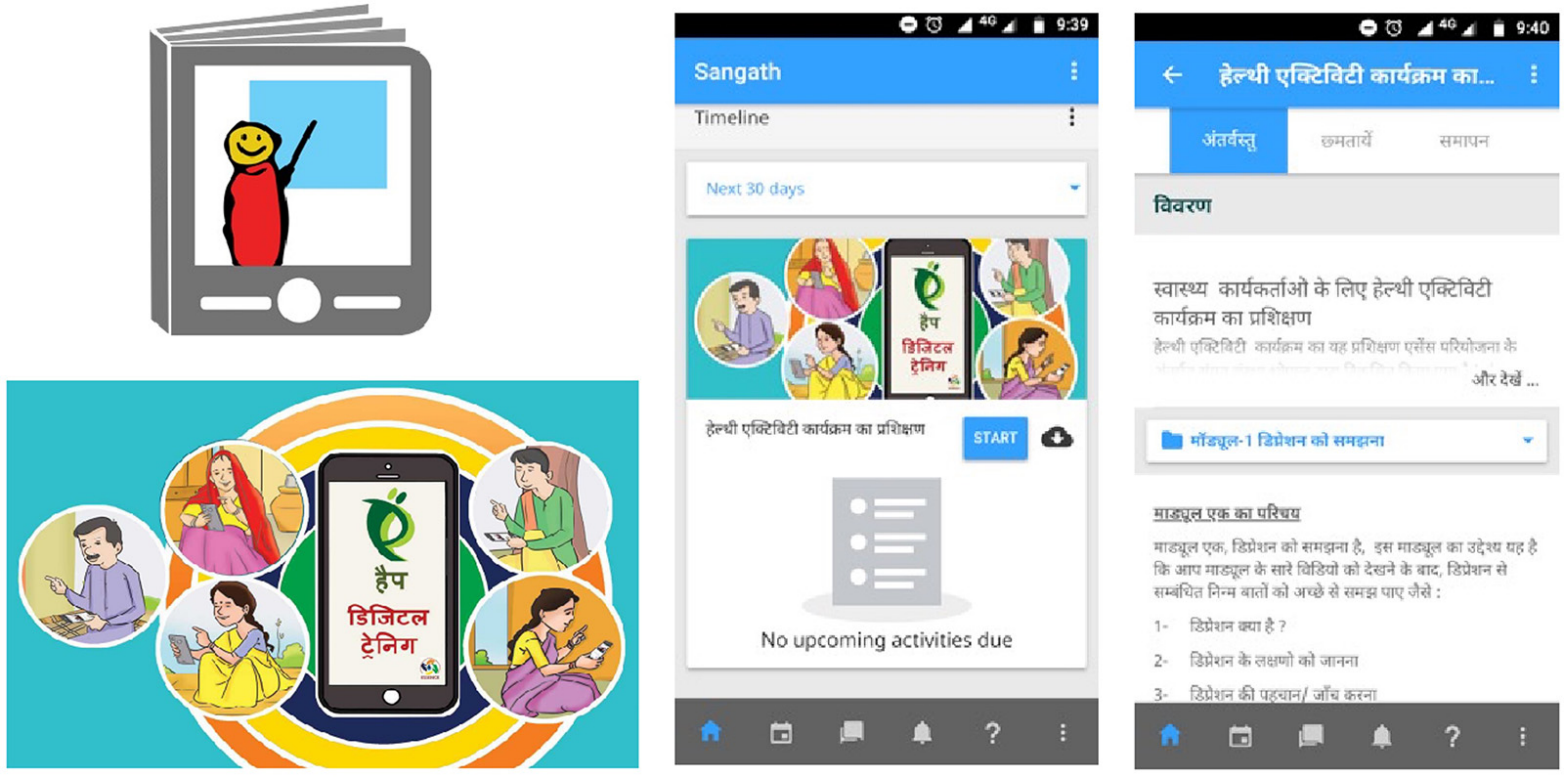
Sample Graphics and Screenshots of the Digital Training Program For a Brief Psychological Intervention for Depression in Rural India

**4. Develop a learning management system (LMS) and upload content**. We worked closely and simultaneously with ASHAs while developing the digital content with an IT agency to develop, test, and finalize the digital LMS platform that housed the training program. The LMS app was chosen based on consultation with ASHA workers in the formative phase of the study, its compatibility with most smartphones (i.e., Android operating system, which is the platform ASHAs are most comfortable with) available in the target setting in rural India, and its ability to function in the event of poor bandwidth and low connectivity.^20^ The LMS consisted of an Android mobile app for easy access to the digital training with offline capabilities to allow access in settings with low bandwidth. The mobile app was also customized using graphics, a user-friendly interface, and text translated into Hindi (Figure 3). We tested the feasibility of the digital training app with a group of volunteers (aged 18–40 years) to address any potential challenges with accessing the training content or technical concerns that may impede navigation within the mobile app. This step allowed for further modification and improvement to the mobile app interface. The key findings from user testing were: (1) the LMS should be simple with basic features and not have complicated, multiple options to navigate the platform; and (2) the interface should have offline accessibility to learn the content to avoid buffering of videos and slow performance due to poor Internet connectivity.

**5. Pilot test the digital training program**. Next, we pilot-tested the digital training program with 33 ASHAs (mean age 34.1, standard deviation=6.75) who had an average of 6.98 years in their current role (standard deviation=4.01) and education level of eighth standard and above.^35^ This step involved user testing the digital training content on the Sangath learning app with ASHAs from primary care facilities in Sehore district, Madhya Pradesh, to replicate a real-world training environment. Smartphones were provided to ASHAs with the downloaded Sangath app, including a SIM card with enough Internet data to access the course and complete the course activities. In alignment with HCD practices to involve end users in the design process, we prioritized ongoing engagement with ASHAs, including in field testing. Through this engagement, we discovered the need to address digital literacy concerns among participants, given that many ASHAs in our user group had no prior experience using a smartphone or completing online training. Therefore, in response to the direct needs of our target population group, we developed a brief digital orientation session to support participants and their use of the smartphone, including instruction in the basic operating features of the device, navigation of the app, and completion of various course activities. These participant insights, which were critical in informing our approach, were also captured through focus group discussions with participants as part of a pilot study.^35^

In alignment with HCD practices to involve end users in the design process, we prioritized ongoing engagement with ASHAs, including in field testing.

### Ethical Approval

All study procedures for this study were approved by institutional review boards at Sangath and Harvard Medical School. Additional Health Ministry Screening Committee approval was obtained from its secretariat at the Indian Council of Medical Research, Government of India.

### Implementation

Our pilot study demonstrated the feasibility, acceptability, and preliminary effectiveness of digital technology for training CHWs to acquire the skills and competencies needed to deliver HAP.^35^ We also evaluated the effectiveness and cost-effectiveness of the digital training in a randomized controlled noninferiority trial in Madhya Pradesh, India.^36^ In the trial, we randomized 340 CHWs from Sehore district to receive either conventional face-to-face training or 2 forms of digital training, which included digital training alone or digital training enhanced with remote coaching support.^37^

Based on our observations from the pilot study, we included the remote coaching support to promote participant engagement and improve successful completion of the training program. The primary outcome of the trial was the change in health workers’ competency to deliver depression care, measured through a validated multiple-choice exam-style competency assessment tool developed as part of the project^38^ and adapted to the local context and language through further engagement of our target group of ASHAs.^39^ In addition, throughout the study, we also collected process indicators, such as the proportion of health workers who successfully completed the training, data metrics related to course progression, digital literacy barriers to navigate the digital content, and any technical challenges that affected the learners’ experience. We also recognized that these training sessions may result in increased workload of health workers. Hence, to explore this potential impact, we collected measures of health worker well-being, such as stress, burnout, and job satisfaction, along with their knowledge, attitudes, and behaviors toward mental health. Since the completion of the trial, we have achieved more than 90% training completion rates among participating health workers, and preliminary analyses have demonstrated that the digital training appeared to be equivalent to conventional in-person face-to-face training when enhanced with remote coaching support.

### Scale-Up

Building on our application of HCD to support the development and evaluation of the digital training platform and as part of broader efforts to scale up interventions for depression, our initiative EMPOWER offers a feasible approach to rapidly scale up the delivery of psychological treatments by providing a sustainable platform to efficiently train CHWs to deliver quality-assured mental health care.^40^ Importantly, drawing on HCD principles, our development and pilot testing of the digital training program involved close collaboration and engagement of ASHAs, our target group of CHWs. Such efforts have ensured that we were able to incorporate their recommendations and perspectives regarding the training content, delivery format, and utility in their daily work routines—all key insights necessary for supporting implementation and scale-up of the training. A key consideration with the use of HCD practices in our context in rural India is that we engaged a group of ASHAs, who were all women, in the design of a training program. These health workers represent the backbone of the primary health care system, experience high work-related burden, and typically have few opportunities for decision-making in their work due to the combination of gender inequality and their location at the bottom of the health system hierarchy. This made our efforts to engage and work with this group of health workers especially important; it helped to amplify their voices and plan for eventual implementation.

Involving ASHAs in the digital training program development and pilot testing ensured that we incorporated their perspectives on training content, delivery format, and utility in daily work.

Our first step toward launching the EMPOWER initiative was to extend this training to 43 CHWs of Jhagadia, a tribal block in selected primary health care settings of Gujarat, another state in India. The training was delivered in the Gujarati language to CHWs with the help of an online LMS nested within a TeCHO+ (Technology Enabled Community Health Operations) platform already in widespread use by the health system across the state. Preliminary results from the pilot study are promising and echo the prior results.^35^ Another component of this initiative involves training more than 1,000 CHWs in 3 districts of Madhya Pradesh, including Narmadapuram, Vidisha, and Raisen, who serve a population of approximately 1.4 million persons. This effort is currently being implemented in partnership with the state government’s health department.

### Lessons Learned

- Formative work employing HCD principles with ASHAs provided crucial information about their preferences for digital content and how local culture is represented.
- When using mobile applications in low- and middle-income countries, it is essential to include offline capabilities to support access in settings with poor bandwidth.
- Iterative feedback cycles with end users, a key aspect of HCD, led to substantial improvements in the product and supported its accessibility, feasibility, and effectiveness for use in the target setting and with the target audience. For example, through field testing, we identified a need to address digital literacy as a core process in the digital training program.
- Leveraging existing technology to support the rollout of the digital training reflects the importance of meeting with stakeholders and following the recommendations and advice of local partners and the health system in which the training will be delivered.
- Digital training is a feasible approach that supports rapid scale-up of training for CHWs to deliver quality-assured mental health care.

Digital training is a feasible approach that supports rapid scale-up of training for CHWs to deliver quality-assured mental health care.

## SUMMARY AND CONCLUSIONS

We presented 2 unique case studies demonstrating the use of HCD in global mental health research in India and the United States. The first case study described early-stage application of HCD, particularly using codesign methodology, to digitally adapt a supportive intervention for resettled refugees. The second case study characterized later-stage application of HCD, engaging end users in the design, testing, and scale-up of a digital training program for a brief psychological intervention for depression. Both case studies draw from projects that reflect the mission and values of the global mental health field and current research priorities identified by the Lancet Commission and other global initiatives, including using task-sharing as the foundation of mental health care delivery, adopting digital tools to facilitate intervention delivery, and implementing community-based interventions. These characteristics are especially beneficial when communities lack an adequate workforce to meet their mental health burden and may help reduce cultural barriers, such as stigma, for those who need treatment.

In line with guidance on best practices for HCD, both programs employed professional designers to develop the media used in each digital application and used participatory design approaches involving end users and iterative feedback loops.^41^ For both case studies, we reveal how HCD can be used to address implementation barriers that contribute to inequities, particularly with regard to digital literacy and equitable access. These strategies included providing devices, adding features for offline use, and, key to the success of both case studies, training for digital use.^42^ Furthermore, design thinking enabled both teams to ensure that their mobile application user interface was user-friendly by presenting material with imagery, animations, and short videos alongside data-light educational information and practice exercises.^43^

Each case study highlighted unique lessons learned. The first case study described how codesign supported community engagement in the development of graphics for a digital application for refugee families participating in the FSI-R in the United States. In particular, this approach improved community buy-in and satisfaction with the components of the interface developed using codesign methodology. We were also able to describe solutions to practical challenges that arose, such as building in support for helping participants acquire new technical skills and managing time constraints when conducting community-based research.

The second case described how HCD principles supported the development, testing, and scale-up of a digital training application for a brief psychological intervention for depression in rural India. Especially notable were its findings that CHWs completed digital training at exceptionally high rates (>90%) and, importantly, that it was as effective as face-to-face training when paired with remote coaching support. These successful findings position the digital training program to be rapidly scaled to train over a thousand CHWs serving over a million people.

These case studies demonstrate the value of employing HCD in research with underserved populations. HCD clearly aligns with the goals of the global mental health field. We demonstrate here how this union between HCD and global mental health can improve user engagement, satisfaction, effectiveness, and scalability. Given these benefits, policymakers, funders, national stakeholders, and others involved in global mental health research should encourage or require participation of end users at all stages of program development, implementation, and evaluation.

## Notes

**Peer Reviewed**

**First published online:** xxxx.
